# Pediatric complex regional pain syndrome: a review

**DOI:** 10.1192/j.eurpsy.2022.1212

**Published:** 2022-09-01

**Authors:** A.M. Matas Ochoa, R. Martinez De Velasco, S. Bravo Herrero, I. Moreno Alonso

**Affiliations:** 1 Hospital Universitario Infanta Leonor, Liaison Psychiatry, Madrid, Spain; 2 Hospital Universitario Infanta Leonor, Psychiatry, MADRID, Spain; 3 Hospital Universitario Infanta Leonor, Psychiatry, Madrid, Spain

**Keywords:** PAIN, COMPLEX, REGIONAL, PSYCHOLOGICAL

## Abstract

**Introduction:**

Complex regional pain syndrome (CRPS) is a chronic localized pain condition that can have a significant impact on the quality of life. It affects children and adolescents as well as adults, but is more common among adolescent girls.

**Objectives:**

To present up-to-date clinical information regarding CRPS in pediatric population.

**Methods:**

A review of recent literature.

**Results:**

In contrast to adults, CRPS appears after an initial event that is typically a minor trauma and occurs more frequently in the lower extremity than in the upper extremity. This syndrome is characterized by spontaneous or stimuli-induced pain, which is disproportionate to the actual incident trauma/stimulus, in the presence of a wide variety of autonomic and motor disturbances. The exact mechanism of CRPS is unknown, although several different mechanisms have been suggested. In many cases, CRPS follows a relatively minor trauma, in some cases, no previous injury was recalled and there are psychological factors implicated. It has been found a high prevalence of anxiety and depression in patients with CRPS and it is considered stress has an important role in inducing or perpetuating CRPS. Standard care consists of a multidisciplinary approach with the implementation of intensive physical therapy in conjunction with psychological counseling; in some patients, pharmacological treatments may help to reduce pain.

**Conclusions:**

A multidisciplinary approach with psychological and psychiatric counseling are needed for effective management of CRPS. Further research in targeting specific mechanisms involved in the pathophysiology of CRPS should lead to prevention of this condition.

**Disclosure:**

No significant relationships.

